# IL-17 in Peritoneal Dialysis-Associated Inflammation and Angiogenesis: Conclusions and Perspectives

**DOI:** 10.3389/fphys.2018.01694

**Published:** 2018-11-26

**Authors:** Janusz Witowski, Julian Kamhieh-Milz, Edyta Kawka, Rusan Catar, Achim Jörres

**Affiliations:** ^1^Department of Pathophysiology, Poznan University of Medical Sciences, Poznań, Poland; ^2^Department of Nephrology, Medical Intensive Care, Charité – Universitätsmedizin Berlin, Berlin, Germany; ^3^Department of Transfusion Medicine, Charité – Universitätsmedizin Berlin, Berlin, Germany; ^4^Berlin Institute of Health, Berlin, Germany; ^5^Department of Medicine I, Nephrology, Transplantation, Medical Intensive Care, University of Witten/Herdecke, Cologne-Merheim Medical Center, Cologne, Germany

**Keywords:** IL-17, inflammation, peritonitis, angiogenesis, fibrosis, VEGF, peritoneal dialysis

## Abstract

Long-term peritoneal dialysis (PD) is associated with peritoneal membrane remodeling. This includes changes in peritoneal vasculature, which may ultimately lead to inadequate solute and water removal and treatment failure. The potential cause of such alterations is chronic inflammation induced by repeated episodes of infectious peritonitis and/or exposure to bioincompatible PD fluids. While these factors may jeopardize the peritoneal membrane integrity, it is not clear why adverse peritoneal remodeling develops only in some PD patients. Increasing evidence points to the differences that occur between patients in response to the same invading microorganism and/or the differences in the course of inflammatory reaction triggered by different species. Such differences may be related to the involvement of different inflammatory mediators. Here, we discuss the potential role of IL-17 in these processes with emphasis on its impact on peritoneal mesothelial cells and peritoneal vascularity.

## Peritoneal Membrane Dysfunction in PD

**FIGURE 1:**
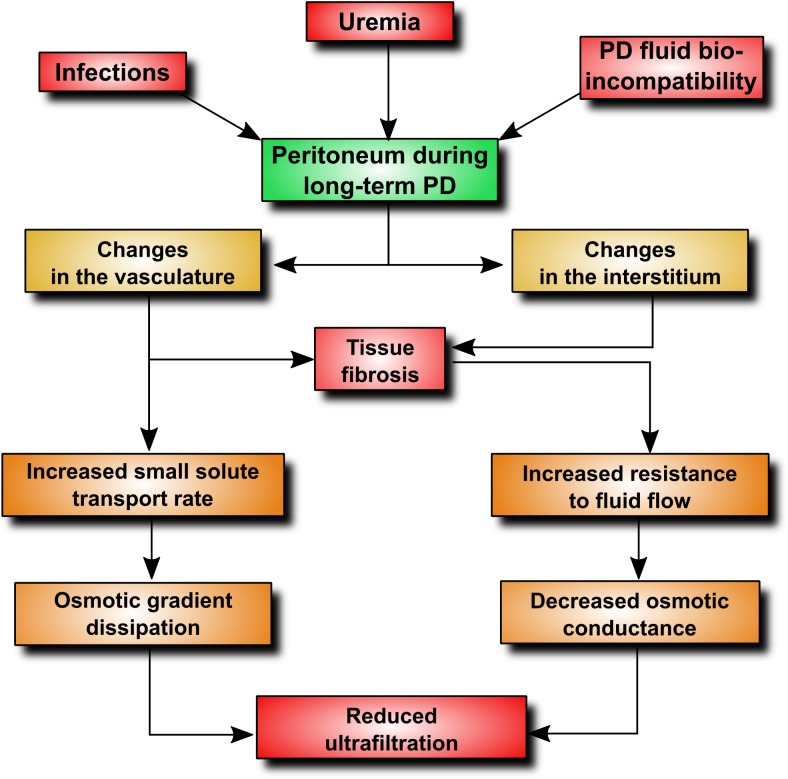
Pathophysiological alterations contributing to ultrafiltration dysfunction during PD. An increase in peritoneal vascularity plays a key role, as it increases vascular surface area available for the transport of small-solutes, including glucose. This leads to an early loss of the glucose osmotic gradient and a decrease in fluid removal. In addition, angiogenesis and adverse vascular remodeling promotes excessive extracellular matrix deposition and tissue fibrosis.

Although peritoneal dialysis (PD) is a well-established treatment modality and the most commonly practiced form of home dialysis, its penetration is well below the utilization rate of 25–30% considered as optimal (Lameire and Van Biesen, 2010). One of the barriers to PD proliferation is the fear that durability of the peritoneum is limited and that the membrane may become unable to sustain treatment at some point. It has been estimated that peritoneal membrane dysfunction is responsible for approximately 30% of all cases of technique failure (Davies et al., 2011). Indeed, longitudinal studies show that peritoneal ultrafiltration gradually decreases with time on PD (Davies et al., 1996). The onset of a decline in ultrafiltration capacity occurs usually 2–4 years after the initiation of PD (Smit et al., 2004) and appears to result from progressive membrane injury and (to some extent) from the loss of residual kidney function. Studies of peritoneal structure and function indicate that two major processes occur during long-term PD treatment: (i) changes in the peritoneal vasculature resulting in increased transport of small solutes, (ii) changes in the peritoneal interstitium leading to reduced osmotic conductance of the membrane (Davies et al., 2011). These processes can be mediated through a number of intertwined pathophysiological mechanisms (Figure 1). By applying an extended 3-pore model of the peritoneum (Davies et al., 2011), it is possible to explain how the peritoneal membrane displays increased transport rates for small solutes and, at same time, becomes more restrictive to water flow. As the transport of small solutes down an osmotic gradient depends largely on area, formation of new blood vessels will increase the surface area available for solute diffusion. On the other hand, fibrotic thickening of the peritoneum will increase resistance to fluid flux and will ultimately decrease water flow through the interstitium. Thus, it appears that the gradual loss of peritoneal ultrafiltration with time is initially related to increased solute transport leading to proportional dissipation of the osmotic gradient. Fibrosis that develops at later stages will then uncouple the osmotic conductance from solute transport resulting in further, and disproportionately severe, reduction in ultrafiltration. In the above scenario, neovascularization plays a key role, both contributing to increased small-solute transport and fuelling fibrosis (Wynn, 2007). Indeed, it has been estimated that up to 75% of patients with ultrafiltration failure may have increased vascular area (Heimburger et al., 1990; Ho-Dac-Pannekeet et al., 1997). Moreover, peritoneal biopsies taken from PD patients show that fibrosis occurs significantly more often in the presence of vasculopathy (Williams et al., 2002), and the density of peritoneal blood vessels and submesothelial and perivascular fibrosis are significantly greater in patients with membrane failure (Mateijsen et al., 1999; Williams et al., 2002). Animal models of PD confirm the existence of inverse correlation between increased vascularization and ultrafiltration (Margetts et al., 2002). These studies also demonstrate that a decline in ultrafiltration can be partially prevented by anti-angiogenic therapy (Margetts et al., 2002).

## Peritoneal Vasculature During PD

Because the alterations in peritoneal vasculature develop in relation to the time spent on PD, their causes are likely to be therapy-related. They may include (i) exposure to PD fluid components, (ii) a progressive decline in residual renal function, and (iii) the occasional episodes of peritonitis. Although acute peritoneal inflammation may cause a profound decrease in ultrafiltration, a single and uncomplicated episode of peritonitis will usually have little long-term effect on the peritoneum (Albrektsen et al., 2004). In contrast, recurrent or clustered episodes of infection with highly pathogenic species may lead to a sustained increase in peritoneal solute transport and a permanent decrease in ultrafiltration (Davies et al., 1996; Wong et al., 2000). This effect is particularly evident in the first year of PD treatment, however, at later stages even patients who never experienced peritonitis may show a similar increase in solute transport rate (Davies et al., 2011). It appears therefore that peritonitis can exacerbate the development of membrane dysfunction over time but it is not the prime and sole determinant of the process.

The components of PD solutions that may be injurious to the peritoneum include non-physiological pH (approximately 5.2), lactate buffer, increased osmolality, and high concentrations of glucose and glucose degradation products (GDPs). A longitudinal analysis has revealed that extensive use of hypertonic PD solutions with high glucose contents precedes an increase in solute transport (Davies et al., 2001). This change in membrane function may lead to less efficient ultrafiltration, which creates a vicious circle by increasing the need for more hypertonic glucose exchanges. These requirements may be further compounded by loss of residual kidney function and decreased urine output. GDPs present in PD fluids in proportion to the concentration of glucose may also contribute to membrane dysfunction by affecting the peritoneal vasculature. It has been demonstrated that GDPs can induce capillary recruitment and vasodilation (Mortier et al., 2002), as well as angiogenesis and hyperpermeability (Hirahara et al., 2006).

The direct effect of uremia on the peritoneal membrane function is less clear. It appears that in many uremic patients some changes in the peritoneum occur even before the start of PD (Williams et al., 2002). Compared with healthy individuals, such patients often have vasculopathy and significant thickening of the submesothelial compact zone (Kihm et al., 2008). These changes are generally attributed to the build-up of uremic toxins, however, their exact nature is poorly defined. The peritoneum of rats made uremic by subtotal nephrectomy shows increased permeability, focal areas of vascular proliferation (Combet et al., 2001), and interstitial fibrosis (De Vriese et al., 2006).

## The Role of Vegf During PD

Vascular endothelial growth factor (VEGF) is a key mediator of pathological changes in blood vessels (Nagy et al., 2007, 2012). Its effect on peritoneal vascularity during PD can be inferred from the association between genetic polymorphisms resulting in increased VEGF production and increased transport rates for small solutes (Szeto et al., 2004). Mesothelial cells are the main source of peritoneal VEGF (Mandl-Weber et al., 2002; Gerber et al., 2006; Boulanger et al., 2007), which can be secreted in response to many stimuli. These are related to both PD fluid exposure and peritonitis (reviewed in Witowski and Jörres, 2011). Expression of the *VEGF* gene is tightly regulated at multiple levels, including transcription, mRNA stabilization, alternative splicing, translation, and subcellular localization (Arcondeguy et al., 2013). Owing to this complexity, the exact molecular mechanism controlling VEGF production during PD is only partially understood. We have previously demonstrated that different cytokines, which are present in the dialysate during peritonitis (e.g., IL-1β, TNFα, TGF-β, and IL-6) can regulate VEGF production by the mesothelium in a context dependent manner by engaging different sets of transcription factors (Catar et al., 2013, 2017).

## Sources and Function of IL-17

**FIGURE 2:**
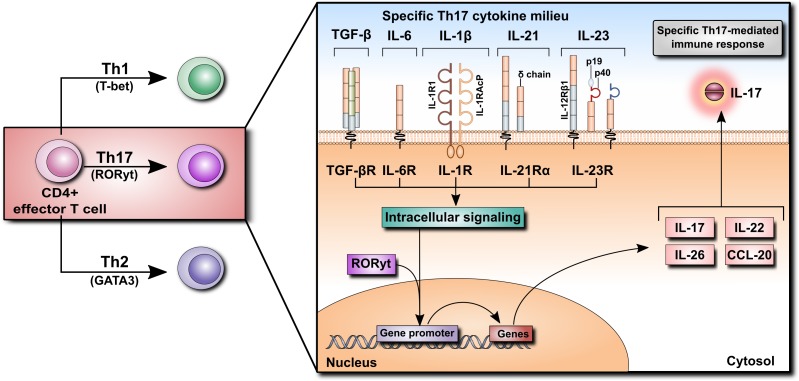
Differentiation of CD4^+^ T cells into Th17 cells. Naïve CD4^+^ T cells can differentiate into Th1, Th2, or Th17 cells according to their specific cytokine milieu. For differentiation of CD4^+^ cells into Th17 cells, TGF-β, IL-6, and IL-1β, as well as IL-21 and IL-23, are required. Cytokine receptor binding initiates an intracellular signaling cascade leading to the translocation of RORyt into the nucleus, inducing the transcription of various cytokines such as IL-17, IL-22, IL-26, and CCL20. Their secretion promotes the Th17-mediated immune response.

The discovery of IL-17 in the 1990s opened a new chapter in immunology. It led to the identification of a distinct type of T helper (Th) cells and shed new light on the role of T cells in inflammation. IL-17 (also known as IL-17A) is the prototypic, the most potent and the best-characterized member of the IL-17 family of cytokines comprising IL-17A through IL-17F (see Beringer et al., 2016 for review). The source of IL-17 has been identified as a subset of CD4^+^ effector T cells that was designated Th17 as it was clearly different from previously known Th1 and Th2 subtypes. It has transpired that naïve CD4^+^ T cells can differentiate into various subsets of effector Th cells (Th1, Th2, and Th17) depending on the exact cytokine milieu. Each Th cell differentiation program is governed by specific transcription factors [T-bet, GATA3 and the retinoic acid-related orphan receptor-γt (RORγt), respectively] and each type of terminally differentiated Th cells produces a specific set of effector cytokines. The polarizing mediators involved at various stages of Th17 cell differentiation include TGF-β, IL-6, IL-1β, IL-21, and IL-23. These combined signals activate the transcription factor ROR-γt, which is required for the production of Th17 cell-specific effector cytokines, including IL-17, IL-22, IL-26, and CCL20 (Figure 2).

**FIGURE 3:**
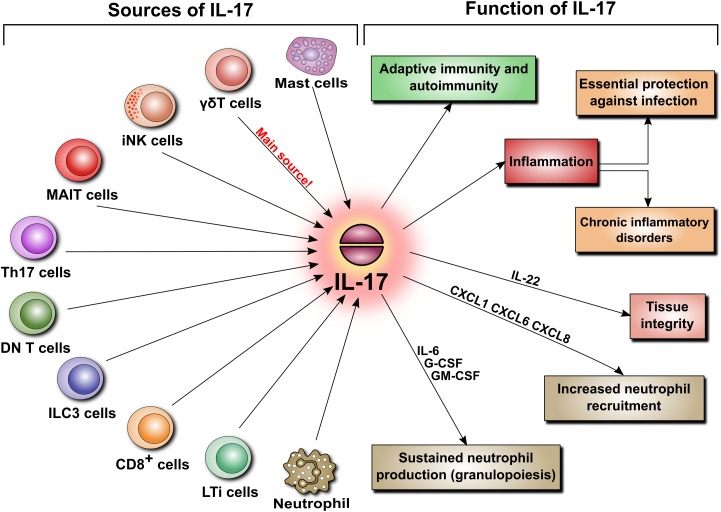
Sources and functions of IL-17. IL-17 secretion is not limited to Th17 cells. When released, IL-17 is involved in various pathophysiological mechanisms. These include adaptive immunity, autoimmunity, and inflammation. The latter is a key element of host defense, but can also lead to chronic inflammatory disorders and tissue remodeling. Additionally, IL-17 regulates tissue integrity and is a potent inducer of neutrophil recruitment and granulopoiesis.

IL-17 can originate not only from Th17 cells but also from innate-like immune cells including CD8^+^ T cells, invariant natural killer T cells (iNKT), lymphoid tissue inducer (LTi) cells, group 3 innate lymphoid (ILC3) cells, CD4^−^CD8^−^-double negative (DN) αβ T cells, and unconventional T cells, such as γδ T cells and mucosal-associated invariant T (MAIT) cells (Figure 3). And it came as a surprise when the main source of IL-17 turned out to be not the expected Th17 cells but γδ T cells, which constitute only a small fraction of lymphocytes. In contrast to naïve Th cells, γδ T cells do constitutively express IL-23R and can immediately respond to IL-23 by secreting IL-17 (Papotto et al., 2017). As IL-23 is derived mainly from sentinel cells such as dendritic cells or macrophages, IL-17-producing γδ T cells can also be viewed as belonging to the category of cells performing surveillance tasks and responding quickly to pathogens.

## Biological Activity of IL-17

By virtue of its ability to induce several cytokines and chemokines, IL-17 has typically been linked to inflammation. In this respect, IL-17 is critically involved both in essential protection against infections and in several disorders characterized by chronic inflammation. By blocking IL-17 signaling in murine models, it has been demonstrated that IL-17 contributes to host defense against extracellular bacterial and fungal pathogens. These include *Klebsiella pneumoniae*, *Staphylococcus aureus*, *Candida albicans*, *Salmonella enterica*, *Streptococcus pneumoniae*, *Listeria monocytogenes*, *Helicobacter pylori*, *Citrobacter rodentium*, and *Trypanosoma cruzi* (see Gu et al., 2013 for review). Here, IL-17 acts mainly as a potent inducer of neutrophil recruitment and granulopoiesis. It does so by promoting the release of chemokines that specifically attract neutrophils (e.g., CXCL1, CXCL6, and CXCL8) and stimulate granulopoiesis in the bone marrow (e.g., IL-6, G-CSF, and GM-CSF). Interestingly, IL-17-producing Th17 cells produce also CCL20 that serves to attract more Th17 cells to the site of inflammation (Figure 3).

Acting together with IL-22, another mediator of sentinel cells, IL-17 contributes also to the maintenance of tissue integrity by enhancing the synthesis of tight junction proteins (claudin) and a number of antimicrobial proteins such as defensins, lipocalin, lactoferrin, and regenerating (REG) and S100A proteins (Cua and Tato, 2010). Moreover, it has been shown that early innate production of IL-17 can influence the generation of antigen-specific Th17 or γδ T cells and contribute to adaptive immunity. Thus generated memory cells persist as long-lived tissue-resident cells, which generate more robust effector responses enhancing pathogen clearance (Lalor and McLoughlin, 2016).

## IL-17 Signaling

IL-17 signaling from the cognate IL-17 receptor has been partially deciphered (reviewed in Gu et al., 2013 and Song and Qian, 2013). It involves the adaptor protein Act1 as evidenced by unresponsiveness of Act1-deficient mice to IL-17 (Qian et al., 2007). Upon IL-17 stimulation Act1 recruits tumor necrosis factor receptor associated factor-6 (TRAF6) that mediates transcription of several target genes through activation of NF-κB and AP-1 transcription factors. In addition, Act1 forms a complex with TRAF5 and TRAF2 to operate at the post-transcriptional level and control mRNA stability (Sun et al., 2011). This IL-17 function is aided by the RNA-binding protein HuR (Herjan et al., 2013). Although Act1 serves primarily as an adaptor protein linking the intracellular domain of the IL-17 receptor with transcription factors (typically of the NF-κB pathway), it has recently been discovered that Act1 itself may exert transcriptional activity by binding to the promoter region of IL-17-responsive genes (Velichko et al., 2016). Although these mechanisms have been found to control the expression of many IL-17-induced cytokines and neutrophil-attracting chemokines (Shen et al., 2006), it is not known whether they are also involved in the regulation of other IL-17 target genes that do not fall into these categories or are expressed in cell types not previously examined.

## IL-17 in the Peritoneum

**FIGURE 4:**
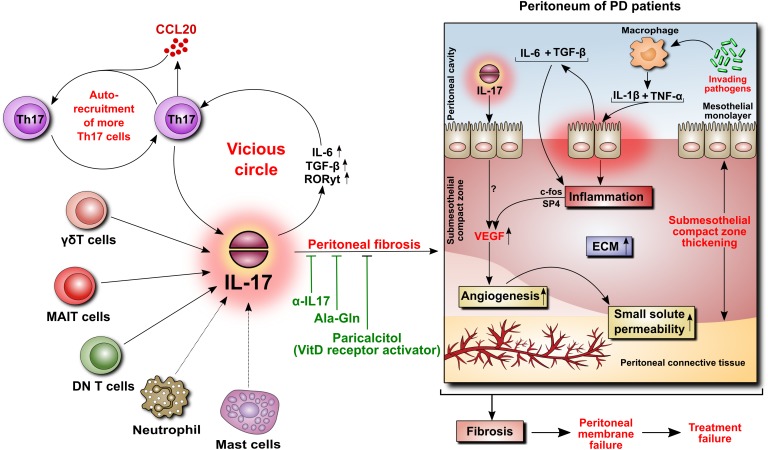
Role of IL-17 in the peritoneum. IL-17 may be critically involved in initiating peritoneal fibrosis. IL-17 is produced primarily by γδT cells and possibly by other innate-like tissue-resident lymphocytes. The increase in IL-17 is associated with increased peritoneal levels of IL-6, TGF-β, and RORγt, leading to the formation of additional IL-17-secreting Th17 cells in a vicious circle. Moreover, Th17 cells release CCL20, a chemokine that boosts the recruitment of further Th17 cells. In the peritoneum of PD patients, IL-17 promotes thickening of the submesothelial compact zone. In addition, inflammation-induced angiogenesis leads to increased small solute transport. In this respect, we previously demonstrated that peritoneal inflammation is linked with angiogenesis through IL-6- and TGF-β-induced VEGF production involving c-Fos and SP4 transcription factors. In rodents, the attenuation of IL-17-mediated responses reduces the extent of peritoneal fibrosis.

IL-17 is virtually undetectable in a healthy human peritoneum, but it can be found in peritoneal biopsies from patients undergoing PD (Rodrigues-Diez et al., 2014). The cells expressing IL-17 were identified as predominantly Th17 cells and γδ T cells, and occasionally as mast cells and neutrophils. The appearance of these cells seemed to correlate with the duration of PD treatment and the extent of tissue fibrosis (Rodrigues-Diez et al., 2014). Using a model of daily PD fluid injections in mice (Gonzalez-Mateo et al., 2009), it has been demonstrated that after 30 days of exposure to PD fluids, but not to control saline, the peritoneum became markedly infiltrated by Th17 and γδ T cells, and its thickness increased in correlation with the levels of IL-17 in the peritoneal cavity (Rodrigues-Diez et al., 2014). Moreover, this increased presence of IL-17-producing cells was associated with increased activity of IL-6, TGF-β, and RORγt, all being instrumental in differentiating Th17 cells. To confirm that IL-17 did indeed contribute to PD fluid-induced alterations, the same experiments were performed in the presence of anti-IL-17 antibodies. These studies showed that the neutralization of anti-IL-17 alleviated the extent of peritoneal fibrosis. Conversely, repeated intraperitoneal administration of exogenous IL-17 led to increased expression of several fibrosis-related genes (fibronectin, TGF-β, α-smooth muscle actin, and fibroblast specific protein-1) and build-up of extracellular matrix (Rodrigues-Diez et al., 2014). Interestingly, a study assessing paricalcitol, a synthetic activator of vitamin D receptor, showed that in the same experimental setting in mice the addition of paricalcitol to PD fluids reduced the extent of peritoneal fibrosis (Gonzalez-Mateo et al., 2014). This effect was attributed partially to inhibition of IL-17-mediated responses as both the numbers of IL-17-producing T cells and the intraperitoneal IL-17 concentrations were significantly reduced (Figure 4).

As indicated earlier, MAIT cells can be another important source of IL-17 (Xiao and Cai, 2017). Like γδ T cells, they accumulate in the peritoneum of patients receiving PD and expand significantly during infections caused by pathogens producing appropriate ligands (Liuzzi et al., 2016). Less clear is the role of IL-17-producing DN T cells in the dialyzed peritoneum. Their contribution to intraperitoneal IL-17 can be surmised from the observation that DN T cells accumulate and secrete IL-17 in the peritoneum of mice infected with *L. monocytogenes* (Riol-Blanco et al., 2010). It will be interesting to see whether DN T cells infiltrate the peritoneum as a result of kidney failure and uremia. In this respect, it has been demonstrated in murine models that DN T cells expand in the kidney after acute ischemia-reperfusion injury (Martina et al., 2016) and are an important subset of IL-17-producing cells in the inflamed kidney (Turner et al., 2012).

It is thought that the differentiation of Th17 cells and their activity during PD can be critically modulated by regulatory T cells (Treg) (Liappas et al., 2015). In this respect, CD69, a membrane glycoprotein induced rapidly on lymphocytes upon activation, has been implicated in promoting Treg development and limiting Th17 differentiation (Martin et al., 2010). In comparison with wild-type mice, the exposure of *cd69*^−/−^ mice to PD fluids for 40 days led to an increase in Th17/Treg ratio and, consequently, to augmented Th17 cell infiltration and increased IL-17 production and peritoneal fibrosis (Liappas et al., 2016). Significantly, exacerbated fibrosis in *cd69*^−/−^ mice could be alleviated by the blockade of IL-17. On the other hand, the effects seen in *cd69*^−/−^ mice could be reproduced in wild-type mice by intraperitoneal administration of CD69-neutralizing antibodies. Similar results were achieved by transplantation of a mixture of bone marrow cells obtained from *Rag2^−/−^_γ_c*^−/−^ double mutant mice and from either *cd69*^−/−^ or wild-type animals. As *Rag2^−/−^_γ_c*^−/−^ mice lack lymphocytes, these were derived only from *cd69*^−/−^ or wild-type mice. This elegant strategy made it possible to demonstrate that CD69 expression in the lymphocytic rather than myeloid compartment of the bone marrow is responsible for controlling Th17 cells (Liappas et al., 2016).

Interestingly, CD69 appears to be constitutively expressed at low levels by tissue-resident memory T (T_RM_) cells and by non-recirculating sessile innate-like lymphocyte subsets, including γδ T cells and MAIT cells (Kimura et al., 2017). The exact role of CD69 expression on these cells is not fully understood, but it appears to be important for cell retention in tissues (Kimura et al., 2017). There is a growing appreciation of the contribution of tissue-resident lymphocytes both to the maintenance of tissue homeostasis and to swift response to infection (Fan and Rudensky, 2016; Gebhardt et al., 2018). In this respect, it has been observed that γδ T cells in mice rapidly produced IL-17 in response to peritoneal infection with *E. coli*, which preceded the influx of neutrophils (Shibata et al., 2007).

It has been proposed that supplementation of PD fluids with the dipeptide alanyl-glutamine (Ala-Gln) could restore an impaired stress response in peritoneal cells and improve peritoneal host defense (Kratochwill et al., 2012, 2016). Indeed, the administration of Ala-Gln to rats and mice treated with PD-fluids markedly reduced the associated peritoneal fibrosis (Ferrantelli et al., 2016). Interestingly, this effect was paralleled by a reduction in peritoneal IL-17 expression and was thus attributed to inhibition of IL-17-driven reactions.

While the above studies clearly documented the peritoneal expansion of IL-17-producing cells in animals infused repeatedly with PD fluids, it remains to be determined, which PD fluid components are responsible for the effect. It has recently been observed that the fraction of IL-17-expressing T cells in peritoneal lavage fluid was greater in mice treated for 8 weeks with a conventional lactate-based PD solution with low pH and high GDP contents than in mice treated with a new neutral-pH low-GDP solution buffered with a mixture of lactate and bicarbonate (Vila et al., 2018). The new solution is viewed as more biocompatible and its use has also been associated with an increase in the dialysate levels of cancer antigen 125 (CA125) (Jones et al., 2001; Fusshoeller et al., 2004; Pajek et al., 2008). As CA125 is thought to reflect mesothelial cells mass (Krediet, 2001), one may hypothesize that less IL-17-mediated inflammation contributes to a better preserved mesothelium.

Clinical PD is frequented by episodes of peritonitis. It has been observed that the effluent concentrations of IL-17 in stable PD patients are very low (typically <5 pg/ml) but increase many-fold at the onset of peritonitis (Lin et al., 2013; Zhang et al., 2017). The magnitude of this increase depends clearly on the class of an invading microorganism; the highest IL-17 levels were recorded during peritonitis caused by Gram-positive bacteria other than streptococci and coagulase-negative staphylococci (e.g., by *S. aureus*) (Zhang et al., 2017). Moreover, it has been reported that patients with a delayed response to seemingly adequate antibiotic treatment had persistently low IL-17 levels (Wang et al., 2011). These observations suggest that IL-17 is an important component of peritoneal host defense. In this respect, it has recently been demonstrated that γδ T cells are the predominant source of IL-17 during *S. aureus*-induced peritonitis in mice (Murphy et al., 2014). Intriguingly, there were two waves of γδ T cell recruited with two distinct γδ T cell subsets involved. An initial rapid influx of Vγ1^+^ and Vγ2^+^ cells was followed by a more sustained infiltration by Vγ4^+^ cells. These Vγ4^+^ cells were retained in the peritoneum and responded by augmented IL-17 production during secondary infection. This led to increased phagocyte recruitment and enhanced bacterial clearance. Accordingly, transfer of *S. aureus*-primed Vγ4^+^ T cells to naïve hosts offered protection against *S. aureus* infection.

On the other hand, it has been observed that extensive peritoneal accumulation of IL-17-producing cells after infection or surgical injury may precede formation of peritoneal adhesions and intra-abdominal abscesses (Chung et al., 2002, 2003). These could be prevented by neutralization of either IL-17 or IL-17-induced CXC chemokines that promote intraperitoneal neutrophil trafficking (Chung et al., 2002). In this respect, we have demonstrated that the peritoneal mesothelium is the main source of CXC chemokines released in response to IL-17 (Witowski et al., 2000). Moreover, IL-17-treated peritoneal mesothelial cells secrete G-CSF that acts to sustain neutrophil production (Witowski et al., 2007). In addition, we have previously demonstrated that mesothelial cells are the main source of intraperitoneal IL-6 (Witowski et al., 1996), which can exert some effects through so-called IL-6 *trans*-signaling (Chalaris et al., 2011). These include selective recruitment of T-cells into the peritoneal membrane (McLoughlin et al., 2005) and maintenance of their Th17 phenotype (Jones et al., 2010).

## IL-17 in Angiogenesis

It has long been suspected that IL-17 may impact on the vasculature, as it can induce CXC chemokines with a characteristic ELR (glutamic acid-leucine-arginine) motif, which are potent angiogenesis promoters (Keeley et al., 2011). These chemokines, including CXCL1, CXCL5, CXCL6, and CXCL8, act via the receptor CXCR2 on endothelial cells stimulating their migration and proliferation. The angiogenic activity of ELR^+^-CXC chemokines has been documented in several animal models of disease, including cancer, corneal neovascularization, and fibrosis (reviewed in Strieter et al., 2007; Keeley et al., 2011, and Santoni et al., 2014). The potential role of IL-17 in angiogenesis was further inferred from the observations that microvessel density in tumors correlated with the number of infiltrating IL-17-producing cells (Numasaki et al., 2003; Zhang et al., 2009; Wakita et al., 2010; He et al., 2011; Meng et al., 2012; Pan et al., 2015a; Huang et al., 2016). Moreover, it has been found that IL-17-transfected cancer cells formed larger and more vascularized tumors when transplanted in mice (Numasaki et al., 2005; Huang et al., 2016), and these effects could be significantly abrogated by the blockade of the CXCR2 receptor. Similarly, an increase in synovial vascularization observed in IL-17-induced arthritis in mice could be reduced by the administration of antibodies against the ELR^+^ chemokine CXCL5 (Pickens et al., 2011).

Less clear is the relationship between IL-17 and VEGF. It has been reported that serum concentrations of IL-17 and VEGF correlate both with each other and with adverse prognosis in patients with colorectal (Liu et al., 2011) and non-small cell lung cancer (Pan et al., 2015a). In this respect, IL-17 has been shown to directly induce VEGF in several malignant cell lines, including gastric (Meng et al., 2012), breast (Amara et al., 2016), and lung cancer (Pan et al., 2015b; Huang et al., 2016), as well as in tumor-associated neutrophils (Benevides et al., 2015). IL-17 can also stimulate VEGF release by normal fibroblasts from the lung, skin, and cornea (Numasaki et al., 2004, 2016; Suryawanshi et al., 2012), by synoviocytes (Honorati et al., 2006; Ryu et al., 2006), and chondrocyte-like cells from the nucleus pulposus (Hu et al., 2016). Such an effect, however, does not seem to be a general phenomenon, as VEGF secretion was not detected in IL-17-stimulated dermal microvascular endothelial cells (Takahashi et al., 2005) and in a number of cancer cell lines (Wu et al., 2016). In the latter, the absence of VEGF induction was attributed to the lack or weak expression of functional IL-17 receptor (Wu et al., 2016).

The exact mechanism of VEGF induction in cells responsive to IL-17 is poorly understood. It appears to be largely cell type-dependent. Few reports presented to date indicate that the regulation of IL-17-VEGF axis may occur via either STAT3- (Pan et al., 2015b; Hu et al., 2016; Wu et al., 2016) or STAT1-controlled (Huang et al., 2016) pathways. Interestingly, it has been suggested that IL-17-stimulated STAT3 activation in some cells required IL-6 induction (Wang et al., 2009). However, in other cells types (e.g., in corneal stromal fibroblasts) IL-17-induced VEGF production did not appear to be related to IL-6 and could not be inhibited by the IL-6 receptor blockade (Suryawanshi et al., 2012). The relationship between IL-17 and VEGF in tumor microenvironment may become even more complex during anti-VEGF therapy. It has been demonstrated that treatment with anti-VEGF drugs leads to an increase in IL-17 in the tumor micro-environment, which initiates a paracrine network that elicits an angiogenic response independently of VEGF and thus contributes to drug resistance (Chung et al., 2013).

## IL-17 and Peritoneal Vasculature

**FIGURE 5:**
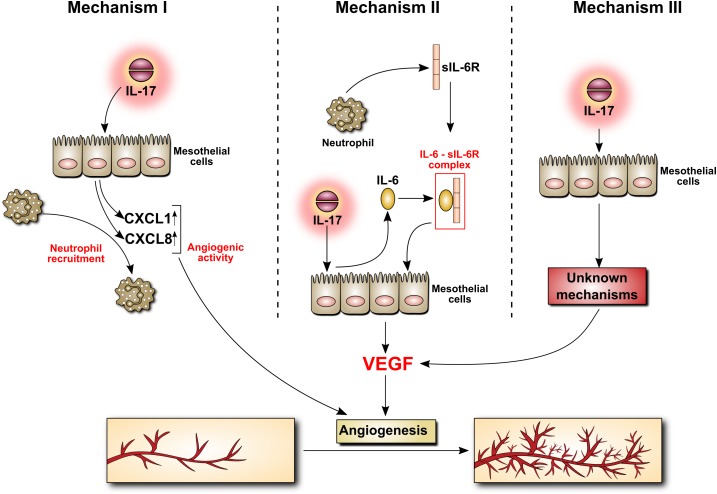
Induction by IL-17 of mediators with angiogenic activity in mesothelial cells. The mechanism behind IL-17 induction of angiogenesis is poorly understood. It may involve the secretion of chemokines with angiogenic activity by mesothelial cells stimulated with IL-17 (Mechanism I). It may also be related to IL-17-induced mesothelial production of IL-6, which may then form a complex with sIL-6R, stimulating VEGF synthesis (Mechanism II). Other, so far unknown scenario(s), cannot be excluded (Mechanism III).

IL-17 can affect peritoneal vasculature through at least three mechanisms, all involving mesothelial cells (Figure 5). Firstly, IL-17 can stimulate mesothelial cells to produce ELR^+^ CXC chemokines such as CXCL1 (GROα) and CXCL8 (IL-8), which – in addition to being powerful neutrophil chemoattractants – do possess angiogenic activity. Secondly, IL-17 can stimulate mesothelial cells to release IL-6. During peritonitis mesothelial cell-derived IL-6 interacts with soluble IL-6 receptor shed from neutrophils and the complex activates mesothelial cells to produce VEGF. Finally, IL-17 can probably directly induce VEGF in mesothelial cells through as yet undefined mechanism.

It is not known what determines the choice of a given pathway *in vivo*. It is probably the presence of a specific combination of cytokines which drives a particular mechanism in mesothelial cells. Not only may such a cytokine cocktail promote differentiation of IL-17-producing cells, but also modulate the effector functions of IL-17. For example, TNFα can synergistically amplify IL-17-induced CXCL1 secretion through both transcriptional and post-transcriptional mechanisms involving stabilization of mRNA transcripts (Sun et al., 2011). Thus, this specific cytokine microenvironment (with IL-17 included) may arise in response to different types of infection and determine the course of inflammation and lead ultimately to changes in peritoneal vasculature.

## Author Contributions

EK, RC, and JW searched the literature and performed the experiments that suggested the mechanisms depicted in Figure 5. JK-M prepared the graphics. JW designed the paper and drafted the manuscript. JW and AJ wrote the manuscript. All authors revised, read, and approved the submitted version.

## Conflict of Interest Statement

The authors declare that the research was conducted in the absence of any commercial or financial relationships that could be construed as a potential conflict of interest.
